# Large-Scale Molecular Simulations on the Mechanical Response and Failure Behavior of a defective Graphene: Cases of 5–8–5 Defects

**DOI:** 10.1038/srep14957

**Published:** 2015-10-09

**Authors:** Shuaiwei Wang, Baocheng Yang, Jinyun Yuan, Yubing Si, Houyang Chen

**Affiliations:** 1 Institute of Nanostructured Functional Materials, Huanghe Science and Technology College, Zhengzhou, Henan 450006, China; 2 Department of Chemical and Biological Engineering, State University of New York at Buffalo, Buffalo, New York 14260-4200, USA

## Abstract

Understanding the effect of defects on mechanical responses and failure behaviors of a graphene membrane is important for its applications. As examples, in this paper, a family of graphene with various 5–8–5 defects are designed and their mechanical responses are investigated by employing molecular dynamics simulations. The dependence of fracture strength and strain as well as Young’s moduli on the nearest neighbor distance and defect types is examined. By introducing the 5–8–5 defects into graphene, the fracture strength and strain become smaller. However, the Young’s moduli of DL (**L**inear arrangement of repeat unit 5–8–5 defect along zigzag-direction of graphene), DS (a **S**lope angle between repeat unit 5–8–5 defect and zigzag direction of graphene) and DZ (**Z**igzag-like 5–8–5 defects) defects in the zigzag direction become larger than those in the pristine graphene in the same direction. A maximum increase of 11.8% of Young’s modulus is obtained. Furthermore, the brittle cracking mechanism is proposed for the graphene with 5–8–5 defects. The present work may provide insights in controlling the mechanical properties by preparing defects in the graphene, and give a full picture for the applications of graphene with defects in flexible electronics and nanodevices.

Graphene, a two-dimensional (2D) material with a single atomic carbon layer array in honeycomb lattice, has attracted widespread interest due to its novel electronic properties^1^, thermal^2^,^3^ and mechanical properties^4^ as well as its tremendous potential applications^5^,^6^,^7^,^8^,^9^,^10^,^11^. Introducing defects and/or vacancies in graphene may alter its properties. Hence, understanding the effect of defects on the fundamental physical properties is necessary and important for applications of graphene in the devices.

An important class of graphene defect, 5–8–5 defect, has been studied both experimentally and theoretically. Through electron bean method, graphene sheets with 5–8–5, 5–8–4–8–5 defects were prepared by Robertson *et al.*^12^. Later, by employing aberration corrected transmission electron microscopy (ACTEM), they investigated the different structural permutations of the tetravacancy defect^13^, and found that the structures depends on the specifics of vacancy creation. By employing scanning transmission electron microscope, Kotakoski *et al.*^14^ identified a significant structural transformation. Chen *et al.*^15^ proposed a method for controlling growth of regular line defect in graphene by means of the electron irradiation. Both the first principles calculations^16^,^17^ and MD simulations^18^,^19^,^20^ are effective means to study the formation and evolution of monovacancy, divacancy, and grain boundaries. Kudin *et al.*^21^ investigated the Raman spectra of graphene sheets with Stone-Wales and 5–8–5 defects using *ab initio* calculations. By employing nonequilibrium molecular dynamics simulations, divacancy was reconstructed by Liang *et al.*^22^, and the main manner for forming that structure is found, i.e. the Stone-Wales transformation. Warner *et al.*^23^ analyzed the bond elongation and the charge density variations in graphene defects using DFT. By using MD simulations, the topology and atomic structure of the defect structures (e.g. 5–8–5 defect) in graphene were examined by Kotakoski *et al.*^24^ Leyssale *et al.*^25^ studied the dynamical behavior of divacancy defect in graphene at high temperatures. Ori *et al.*^26^ prepared 5–8–5 defects using isomeric modifications of graphene mesh. In the previous studies, little is known about the mechanical properties and failure behavior for graphene sheet with 5–8–5 defects.

In this paper, a family of the graphene with 5–8–5 defects are designed and prepared. Then, systematically detailed studies on the mechanical responses of graphene with various 5–8–5 defects are examined. The dependence of ultimate strength, ultimate strain and Young’s modulus of graphene with defects on the nearest neighbor distance between defects and defect types is determined. A possible brittle cracking mechanism was proposed: i.e. the failure of graphene with 5–8–5 defects can initiate from either the bond shared by the 5–8 rings or the bond shared by 6–8 rings, depending on the defect arrangement as well as the loading orientation.

## Model and Methodology

**Figure 1:**
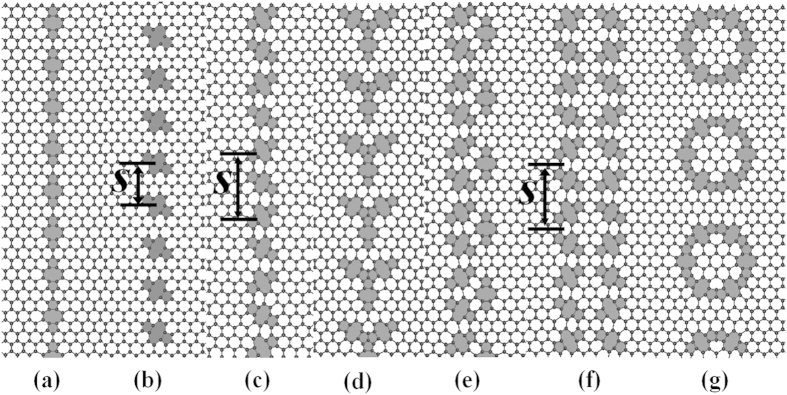
The designed 5–8–5 defects in a graphene membrane. (**a**) DL: **L**inear arrangement of repeat unit 5–8–5 defect along zigzag-direction of graphene; (**b**) DS: a **S**lope angle between repeat unit 5–8–5 defect and zigzag direction of graphene; (**c**) DZ: **Z**igzag-like 5–8–5 defects; (**d**) DY: A repeat unit of **Y**-like 5–8–5 defects; (**e**) DT: A repeat unit of **T**riangular 5–8–5 defects; (**f**) DQ: a repeat unit of **Q**uadrangular 5–8–5 defects; (**g**) DH: a repeat unit of **H**exagonal 5–8–5 defects. The nearest neighbor distance *s* refers to the distance between the two centers of mass of two nearest neighbor repeat units.

Graphene with six different kinds of 5–8–5 defects are prepared and presented in Fig. 1. They are: DL (**L**inear arrangement of repeat unit 5–8–5 defect along zigzag-direction of graphene), DS (a **S**lope angle between repeat unit 5–8–5 defect and zigzag direction of graphene), DZ (**Z**igzag-like 5–8–5 defects), DY (A repeat unit of **Y**-like 5–8–5 defects), DT (A repeat unit of **T**riangular 5–8–5 defects), DQ (a repeat unit of **Q**uadrangular 5–8–5 defects) and DH (a repeat unit of **H**exagonal 5–8–5 defects). The size of the graphene is approximately 20 × 20 (nm)^2^. The adaptive intermolecular reactive bond order potential (AIREBO)^27^, which was confirmed to reproduce the mechanical properties of graphene sheet^28^,^29^ and the bond breaking and forming^30^ very well, was used for the interaction between carbon atoms. Following Wei’s work^31^, the C-C bond cutoff distance of 1.92 Å, which can avoid high bond forces and nonphysical results before fracture occurs, is employed.

Simulations were performed by employing Larger-scale Atomic/Molecular Massively Parallel Simulator (LAMMPS)^32^. Periodic boundary conditions (PBCs) are employed in all the three dimensions. The initial configuration is relaxed to the equilibrium state with a time step of 1.0 fs. The equilibrium is realized by a conjugate-gradient algorithm, and then a simulation with isothermal-isobaric (NPT) ensemble is carried out for 10 ns to eliminate the inner stress of graphene with defects, followed by the simulation with microcanonical ensemble (NVE) for another 10 ns. After the system approaches to equilibrium, the tension load is applied to the graphene sheet in the armchair or zigzag direction with a strain rate of 0.0004 ps^−1^ and the increment every 1 ps.

The stress on each carbon atom is calculated via the following virial stress equation^33^,^34^:


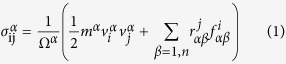


In equation (1), the *α* and *β* are the indices of stress tensors along the Cartesian coordinate axis, *i* and *j* are the atomic positions, Ω^*α*^, *v*^*α*^ and *m*^*α*^ are the atomic volume, velocity and mass, respectively. *r*^*j*^ and *f*^* i*^ are the distance and force. In addition, the thickness 3.4 Å is employed to compute the atomic stress in graphene sheet^35^. The stress is averaged over all the atoms in the entire graphene sheet every 0.01 ns. The calculation of the Young’s modulus, the fracture strain, the fracture strength, tensile strength, and ultimate strain are following references^30^,^36^,^37^.

## Results and Discussion

**Figure 2:**
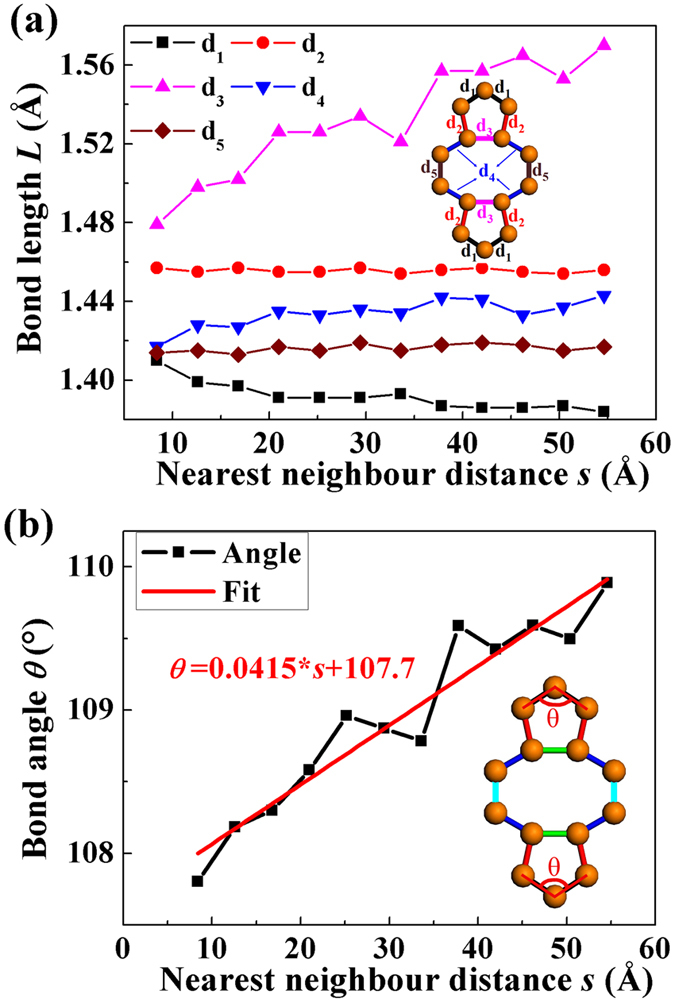
Bond length and bond angle. (**a**) The average bond length *L* and (**b**) bond angle *θ* after relaxation as functions of the nearest neighbor distance *s* in a graphene with DL defect. d_1_, d_2_, d_3_, d_4_, and d_5_ refer to five different types of bonds.

Figure 2 shows the average bonds lengths and bond angles as functions of the nearest neighbor distance *s* (refers to the distance between the two centers of mass of two nearest neighbor repeat units, see Fig. 1) for the DL defect. Five different types of bonds in 5–8–5 defects (see Fig. 2a) are taken into account. For a selected *s*, d_3_ > d_2_ > d_4_ > d_5_ > d_1_ (Fig. 2a). For example, when selected *s* = 8.4 Å, the average bond lengths, d_3_ = 1.479 Å > d_2_ = 1.457 Å > d_4_ = 1.417 Å > d_5_ = 1.414 Å > d_1_ = 1.410 Å. Additional, with *s* increases, the bond lengths d_3_ and d_4_ increase significantly and d_1_ decreases, while d_2_ and d_5_ are not sensitive to *s*. For instance, the d_3_ (1.57 Å) at *s* = 54.6 Å is 6% higher than that when *s* = 8.4 Å. The d_1_ (1.384 Å) at *s* = 54.6 Å is 2% lower than that when *s* = 8.4 Å. Our results of the average bond lengths are in excellent agreement with the experiment data and DFT results for the two 5–8–5 defects^23^. From the experiment, the d_1_, d_2_, d_3_, d_4_ and d_5_ are 1.33–1.45 Å, 1.30–1.49 Å, 1.53–1.67 Å, 1.43–1.46 Å, and 1.31–1.53 Å, respectively. Figure 2b shows the average angle *θ* as a function of the nearest neighbor distance (*s*) for the graphene with DL defect. The average angle *θ* changes monotonically with the increase of *s. θ* and *s* can be fitted by a linear function, that is, *θ* = *ks* + *b*. Using the least-squares technique, the fitting parameters are obtained, that is, *θ* = 0.04115 * *s* + 107.7 (see Fig. 2b).

**Figure 3:**
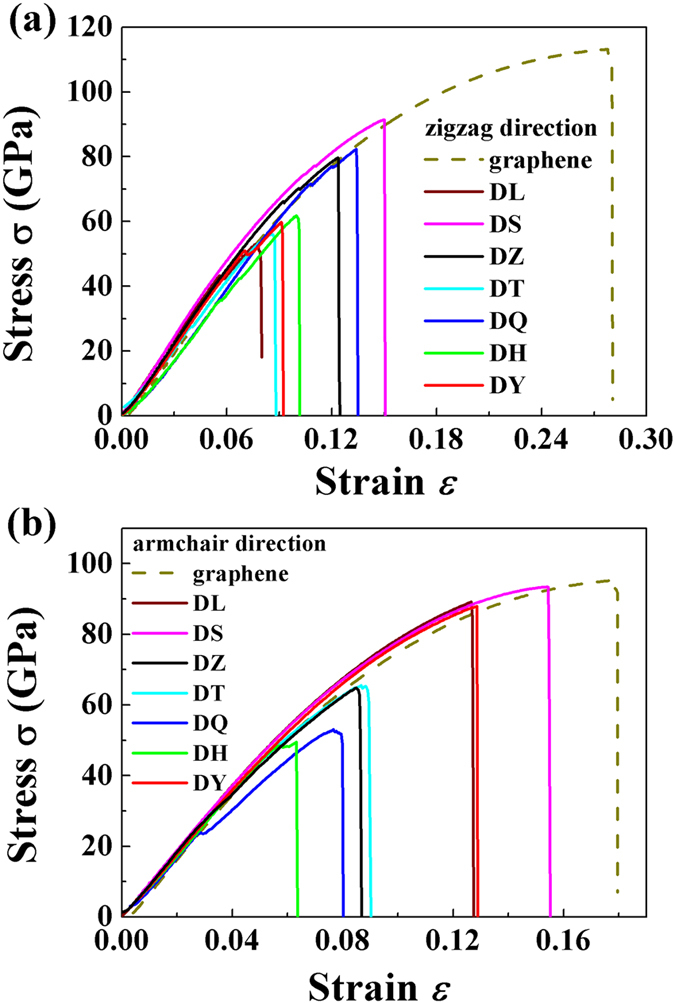
Stress-strain curves. The stress-strain curves of pristine graphene and graphene with various defects (the shortest *s* for each structure) under loading in the zigzag (**a**) and armchair (**b**) directions.

The stress-strain curves of pristine graphene and graphene with various 5–8–5 defects (the shortest *s* for each structure) are presented in Fig. 3. With increasing strain, the stress of graphene with defects increases nonlinearly and then instantaneous decreases. The instantaneous decreases occurs because of bond(s) failure. The behavior of the stress-strain curves for graphene with defects is similar as that from the pristine graphene. The fracture strength and fracture strain for these defects are lower than those from pristine graphene. For example, the fracture strength for DL defect is 52 GPa in the zigzag direction, which is lower than that (113 GPa) for pristine graphene in the same direction. The fracture strain 0.063 for DH defect in the armchair directions is approximately 64% lower than that (0.177) for pristine graphene in the same direction.

Based on Fig. 3, one can calculate that the Young’s modulus of pristine graphene are 772.19 and 962.79 GPa for zigzag and armchair directions, respectively. The results agree with the data for zigzag from previous MD simulations^38^. This behavior indicates that the methodology adopted can provide a reasonable description on the mechanical response.

**Figure 4:**
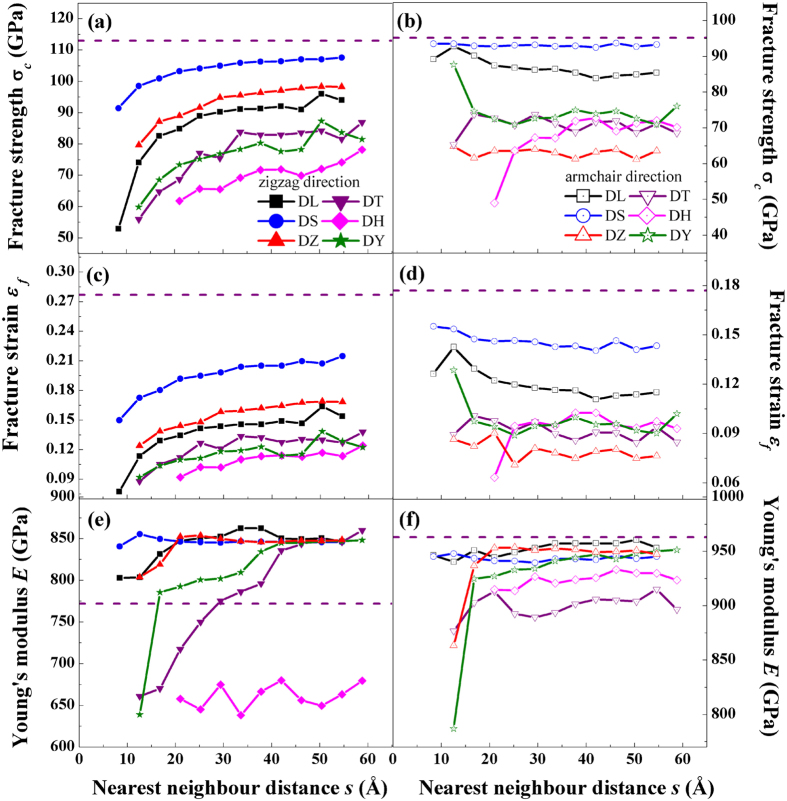
The mechanical properties of graphene with various defects. (**a**,**b**) The fracture strength (*σ*_*c*_) versus the nearest neighbour distance (*s*); (**c**,**d**) The fracture strain (*ε*_*f*_) versus *s*. (**e**,**f**) The Young’s modulus (*E*) versus *s*. The solid (left) and open (right) symbols represent the loading direction in zigzag and armchair, respectively. The dash lines indicate the corresponding data of pristine graphene.

Figure 4 displays the fracture strength, fracture strain and Young’s modulus versus the nearest neighbour distance (*s*) of graphene with various defects. As *s* increases, the fracture strength and strain in the zigzag direction first increase and then tend to constants. The fracture strength and strain of DH in the armchair direction first increase and then tend to constants as *s* increases while those of DL, DS, DZ, DT, and DY are not sensitive to the nearest neighbor distance *s*. For a selected graphene or graphene with defects, the fracture strength and fracture strain of zigzag oriented are larger than those of armchair oriented, while the Young’s moduli in the armchair direction are much higher than those in zigzag direction.

From Fig. 4, one can found that, by introducing 5–8–5 defects in graphene, the fracture strength and fracture strain become smaller (comparing to the pristine graphene, see Fig. 4a–d). Similar results have been reported in one grain boundary (one-GB)^27^ and Stone-Thrower-Wales (STW) defects^29^,^30^ cases by using MD simulations. Comparing with pristine graphene, the Young’s moduli of graphene with defects in the armchair direction become smaller (see Fig. 4f). However, the Young’s moduli of DL, DS, and DZ defects in the zigzag direction become larger than those from pristine graphene, while Young’s modulus of DH become smaller. The Young’s moduli in the zigzag of DT and DY increase as *s* increases. Comparing with Young’s modulus of pristine graphene, the Young’s moduli of defects DT and DY in the zigzag direction become smaller with small *s*, and become larger with large *s* (see Fig. 4e). For instance, the Young’s moduli of all the defects in the zigzag direction except DH trend to be stabilized with *s* > 40 Å. The average Young’s moduli is approximately 846 GPa, which is 9.6% higher than that of pristine graphene (772 GPa). The maximum Young’s modulus of 863 GPa is obtained, which is 11.8% higher than that of pristine graphene. The average Young’s moduli are larger than those of graphene containing one-GB^27^(771–810 GPa) and with STW^36^,^38^ defects (768–784 GPa) in previous MD simulations.

**Figure 5:**
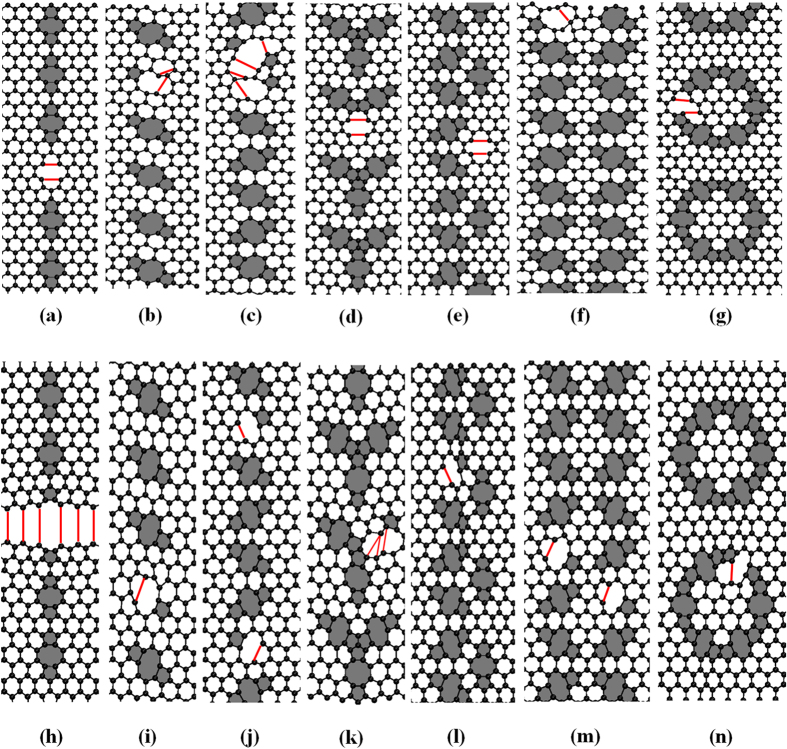
Conformations after the fracture occurs. (**a**–**g**) Tension loading in the zigzag direction. (**h**–**n**) tension loading in the armchair direction. The red solid lines represent the bond connection states before cracking. The pentagons and octagons are colored in light grey.

To identify the failure mechanisms of graphene with defects, conformations of graphene with defects after the fracture occurs are shown in Fig. 5. For each case, the first breaking bond is either shared by 5–8 rings or by 6–8 rings. After that, a hole is generated from the origin of the crack for the further stretching.

**Figure 6:**
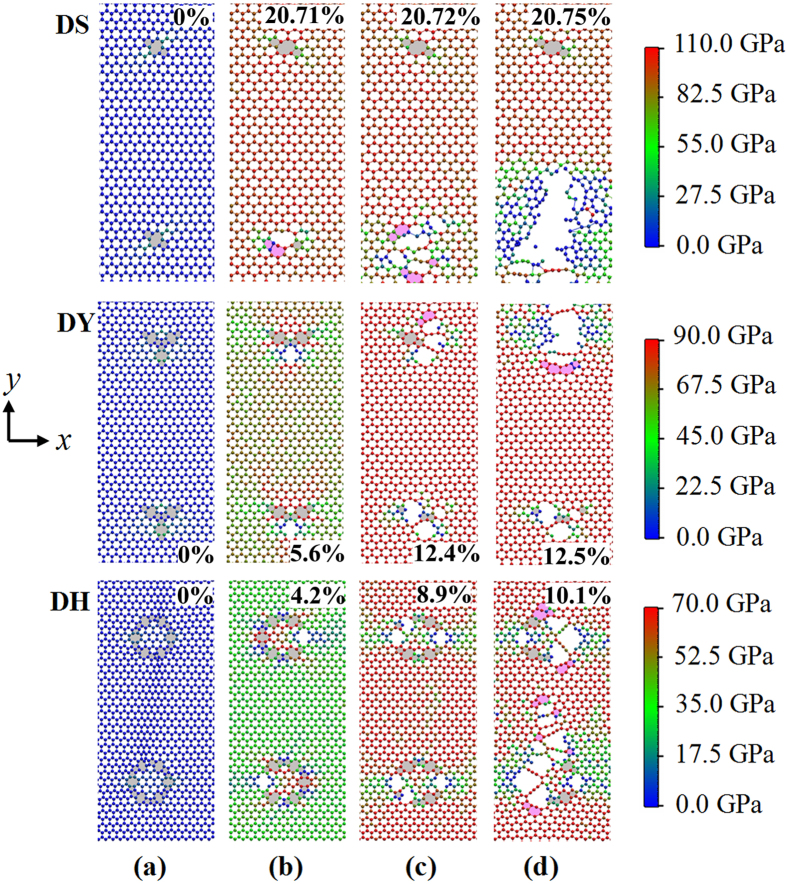
Deformation processes and stress field distribution of a graphene with DS, DY and DH defects when the nearest neighbor distance *s* = 46.2 Å. The loading direction is in the zigzag direction (*x* axis in this work). (**a**) after relaxation (**b**) bonds break (**c**) more bonds break (**d**) nanowires were generated. The original pentagons and octagons are colored in light grey, and the new formed pentagons and heptagons are colored in pink. Strain values displayed in the top right corner or the bottom corner.

In order to present the fracture process of graphene sheet with defects, the atomic stress distributions for DS, DY and DH defects with the nearest neighbor distance *s* = 46.2 Å are illustrated in Fig. 6. The loading direction is in the zigzag direction. After relaxing, the graphene sheet with DS, DY or DH defect changes from its original flat configuration to a wavy configuration. As the simulation time increases, the C-C bonds which shared by 5–8 rings crack at strain 20.71%, 5.6% and 4.2% for DS, DY and DH defects, respectively (see Fig. 6b). This is due to the atoms shared by 5–8 rings bear much higher stress than other atoms. After the bond cracks, a crack blunting behavior for DY and DH defects occurs. Later, more bonds shared by 5–8 rings break with the further stretching (see Fig. 6c). Cracks are rapidly generated around the breaking bonds. More short nanowires can be observed in the Fig. 6d. These cracks are along the defect line and normal to the loading direction. Similar results are also found in graphene with grain boundaries, but the fracture process started from the bonds shared by 7–6 rings at the grain boundary^39^,^40^. From Fig. 6, one can see that the bonds break directly and no Stone-Wales transformation occurs. This is because that the system could not overcome the Stone-Wales transformation barriers at low temperature cases. Hence, the brittle fracture will dominate the fracture process in this mode. This finding is consistent with the previous MD simulations on carbon nanotubes^41^,^42^,^43^ and graphene GBs^40^,^44^. The ductile fracture might be occurred by raising temperatures.

**Figure 7:**
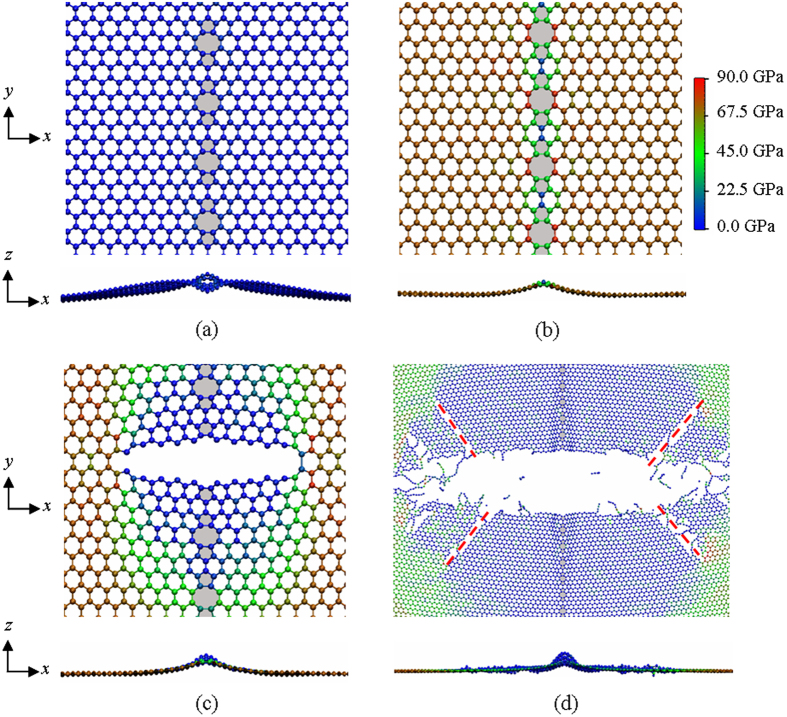
Stress field distribution and deformation processes of a graphene with DL defect with the nearest neighbor distance *s* = 8.4 Å when tension loads in the armchair direction (*y* axis in this work). (**a**) the initial equilibrated stage (**b**) at 12.2% strain (**c**) bonds break at 12.4% strain (**d**) completely broken stage. The pentagons and octagons are colored in light grey. The red dashed lines indicates the rifts along the zigzag direction.

It should be mentioned that most of previous papers are regarding the loading direction perpendicular to the defect line, but less consider the tensile in the parallel to the defect line. The deformation process of DL defect pulled in the armchair direction are illustrated in Fig. 7. After relaxing, the graphene sheet transforms from its original smooth flat state to a wrinkle along the *z* axis (Fig. 7a). This is due to the existence of 5–8–5 defects. As the simulation time increases, the atoms/rings wrinkled move upward along the defect line and the bonds shared by 6–8 rings bear much higher stress than other atoms (see Fig. 7b). After that, a crack is rapidly generated around those bonds (Fig. 7c) along the zigzag direction, i.e. the crack propagates normal to the loading direction. In this simulation, the fracture pattern is also brittle fracture. Similar results were observed on graphene sheet with cracks via experiments^45^,^46^ and graphene sheet via MD simulations^47^,^48^. From Figs 5, 6, 7, one can conclude that the failure of graphene with 5–8–5 defects was dominated by the brittle cracking mechanism.

## Conclusion

A family of graphene with 5–8–5 defects were prepared and their mechanical properties were examined by using molecular dynamics simulations. Dependence of fracture strength and strain as well as Young’s moduli (in both zigzag and armcharm directions) on the nearest neighbor distance and defect types was investigated. The fracture strength and strain in the zigzag direction first increase and then tend to constants with the nearest neighbor distance increases. For a selected graphene or graphene with defects, the fracture strength and fracture strain of zigzag oriented are larger than those of armchair oriented. The Young’s moduli in the armchair direction are much higher than those in zigzag direction. The fracture strength and fracture strain become smaller by introducing 5–8–5 defects in graphene. Comparing with pristine graphene, the Young’s moduli of graphene with defects in the armchair direction become smaller, whereas the Young’s moduli of DL, DS, and DZ defects in the zigzag direction become larger. The brittle cracking mechanism is proposed for the graphene with 5–8–5 defects, and the first breaking bond is either shared by 5–8 rings or by 6–8 rings.

## Additional Information

**How to cite this article**: Wang, S. *et al.* Large-Scale Molecular Simulations on the Mechanical Response and Failure Behavior of a defective Graphene: Cases of 5-8-5 Defects. *Sci. Rep.* **5**, 14957; doi: 10.1038/srep14957 (2015).
